# Aging Policy issues of Concern to Older Adults: A Case Study of Older Adults Volunteer Team in Taiwanese Hospital

**DOI:** 10.1093/geroni/igaf122.2471

**Published:** 2025-12-31

**Authors:** Liying Chen, Tsuann Kuo

**Affiliations:** Taipei City Hospital Renai Branch, Taipei, Taipei, Taiwan (Republic of China); Chung Shan Medical University, Taichung, Taichung, Taiwan (Republic of China)

## Abstract

In 2021, Taiwan implemented the Aged-Society White Paper, which consisted of eleven domains including health and medical care, long-term care, social participation and learning, age discrimination, age-friendly environment, intergenerational learning, artificial intelligence technology, family and community, social security system, and research on aging society issues. However, there is limited research on the level of awareness and concerns about these issues among the older adults. . The purpose of this research was to explore the issues of concerns among hospital volunteers on the eleven domains from the Aged-Society White Paper actions. A Likert scale-based questionnaire was designed and data were analyzed to find out whether there are differences in the level of interest among different domain topics across different demographic variables. A total of 138 volunteers participated and the average age was 75. The results showed that the top concerns for older adults were health and medical care, followed by long-term care, and artificial intelligence technology. Significant differences were found in the artificial intelligence technology issue related to age. Significant differences were found in all issues related to education except long-term care, artificial intelligence technology, and research on aging society issues. Education and age were significant determinants in contributing to volunteers’ awareness and interests. Furthermore, the implications for why the top issues can be expanded to older adults should be investigated. It is crucial to focus on the diversity of older adults and identify other Aged-Society policies that require further promotion across the life course.

